# Parabrachial Neurons Promote Behavior and Electroencephalographic Arousal From General Anesthesia

**DOI:** 10.3389/fnmol.2018.00420

**Published:** 2018-12-04

**Authors:** Tianyuan Luo, Shouyang Yu, Shuang Cai, Yu Zhang, Yingfu Jiao, Tian Yu, Weifeng Yu

**Affiliations:** ^1^Department of Anesthesiology, Renji Hospital, School of Medicine, Shanghai Jiao Tong University, Shanghai, China; ^2^Guizhou Key Laboratory of Anesthesia and Organ Protection, Zunyi Medical College, Zunyi, China

**Keywords:** parabrachial neurons, arousal, propofol, isoflurane, anesthesia, fiber photometry

## Abstract

General anesthesia has been used clinically for more than 170 years, yet its underlying mechanisms are still not fully understood. The parabrachial nucleus (PBN) in the brainstem has been known to be crucial for regulating wakefulness and signs of arousal on the cortical electroencephalogram (EEG). Lesions of the parabrachial complex lead to unresponsiveness and a monotonous high-voltage, and a slow-wave EEG, which are the two main features of general anesthesia. However, it is unclear whether and how the PBN functions in the process of general anesthesia. By recording the levels of calcium *in vivo* in real-time, we found that the neural activity in PBN is suppressed during anesthesia, while it is robustly activated during recovery from propofol and isoflurane anesthesia. The activation of PBN neurons by “designer receptors exclusively activated by designer drugs” (DREADDs) shortened the recovery time but did not change the induction time. Cortical EEG recordings revealed that the neural activation of PBN specifically affected the recovery period, with a decrease of δ-band power or an increase in β-band power; no EEG changes were seen in the anesthesia period. Furthermore, the activation of PBN elicited neural activation in the prefrontal cortex, basal forebrain, lateral hypothalamus, thalamus, and supramammillary nucleus. Thus, PBN is critical for behavioral and electroencephalographic arousal without affecting the induction of general anesthesia.

## Introduction

General anesthesia has been used during surgery for more than 170 years, yet the underlying mechanisms of the anesthetic agents used daily in operating rooms are not completely understood. There has been a growing appreciation that neural pathways regulating the endogenous sleep–wake systems are involved in the induction of, maintenance of, and recovery from general anesthesia (Zecharia et al., 2009; Mashour and Pal, 2012). Therefore, research investigating the mechanisms of general anesthesia has always focused on sleep–wake systems. Pharmacological modulation of cholinergic (Tai et al., 2014; Kenny et al., 2016), histaminergic (Luo and Leung, 2011), orexinergic (Kelz et al., 2008; Zhang et al., 2016), noradrenergic (Vazey and Aston-Jones, 2014), or dopaminergic (Taylor et al., 2013; Zhou et al., 2015) arousal pathways have demonstrated that endogenous sleep–wake pathways contribute to the regulation of the recovery process after anesthesia. However, recent evidence indicates that these systems play only a modulatory role in sleep, as lesions of these systems have little impact on daily sleep–wake intervals or EEG patterns (Blanco-Centurion et al., 2007; Fuller et al., 2011). Instead, the key sleep–wake regulatory system may depend upon the function of the fast neurotransmitters glutamate and GABA (Saper and Fuller, 2017), and these may modulate the recovery process after anesthesia as well.

The parabrachial nucleus (PBN) in the dorsolateral pons contains a large population of glutamatergic neurons, and sends extensive projections to the cerebral cortex, basal forebrain (BF), hypothalamus, and thalamus (TH; Qiu et al., 2016), and is a key site in the ascending arousal system (Fuller et al., 2011). PBN is indispensable in generating and maintaining EEG arousal and behavioral wakefulness (Fuller et al., 2011). Lesions of the lateral and medial PBN result in a coma-like state with a monotonous (<1.0 Hz) EEG and behavioral unresponsiveness, reminiscent of general anesthesia. In addition, stimulation of the pontine PBN using the designer receptors exclusively activated by designer drugs (DREADDs), potently drives cortical arousal and behavioral wakefulness via the BF and the lateral hypothalamus (LH; Qiu et al., 2016), which are two areas that modulate sensitivity to anesthetics (Luo and Leung, 2009; Leung et al., 2011; Zhang et al., 2012). Furthermore, genetically deleting glutamate signaling in the PBN leads to a large reduction in total wakefulness and an increase of delta oscillations in the waking EEG (Kaur et al., 2013), which, to some extent, is similar to the EEG under general anesthesia—with globally coherent slow-wave activity in the delta frequency range (Molaee-Ardekani et al., 2007). Importantly, it has also been reported that electrically stimulating the PBN nucleus induces reanimation from isoflurane general anesthesia (Muindi et al., 2016). This cumulative evidence suggests that the PBN plays a critical role in promoting and maintaining wakefulness. However, there is little evidence about the effect of PBN neurons on the anesthesia–arousal circle after propofol administration. It is unknown whether the activation of PBN could block the onset of anesthesia by isoflurane and propofol anesthesia in rodent models or the clinical setting.

Our hypothesis for this study was that PBN neurons are suppressed during either propofol or isoflurane anesthesia, while they are re-activated during the recovery period and contribute to the restoration of awareness. To test this, we employed real-time calcium fiber photometry (Guo et al., 2015), a very sensitive method, to measure the PBN activity in the process of propofol and isoflurane anesthesia. We also used DREADDs to activate the PBN neurons (Qiu et al., 2016). We found that PBN activity was inhibited in the anesthesia phase, yet was dramatically activated in the recovery period. DREADDs activated PBN neurons and shortened the recovery period without affecting the induction time. We further analyzed EEG changes after the activation of PBN and found that it did not change the EEG during anesthesia but drove cortical arousal from general anesthesia. Finally, we analyzed projection areas from the PBN and found that several arousal-related areas were activated, suggesting that an arousal network is activated following the activation of PBN. These findings show that PBN plays a functional role in modulating arousal but not in the induction process of general anesthesia.

## Materials and Methods

### Animal Care and Use

All experimental procedures were approved by the animal care and use committees of Zunyi Medical University, Guizhou, China, and followed the Guide for the care and use of laboratory animals in China (No. 14924, 2001). Pathogen-free, adult, male Sprague-Dawley rats (weight 280–320 g at surgery, *n* = 42) were purchased from the animal center of Third Military Medical University (license number: SCXK2012-0005; Chongqing, China). Ten rats were used in the fiber photometry experiment (5 rats for propofol, and 5 rats for isoflurane), 32 rats were used in the DREADDs experiment (16 rats for propofol, and 16 rats for isoflurane; in each group, 8 rats for hM3Dq-EGFP, and 8 rats for EGFP). Twelve rats with hM3Dq-EGFP in the DREADDs experiment were implanted with EEG electrodes (6 rats for propofol, and 6 rats for isoflurane). Rats were housed in an ambient temperature of 22 ± 0.5°C with a relative humidity 60 ± 2% on an automatically controlled 12-h light/12-h dark cycle (light on at 8:00 am). Food and water were provided *ad libitum*.

### Calcium Fiber Photometry

Rats (*n* = 10) were anesthetized with chloral hydrate (7% in saline, 400 mg/kg, i.p.) and then placed on a stereotaxic apparatus (RWD Life Science, Shenzhen, China). Lidocaine (2%) was subcutaneously injected for local anesthesia before exposing the surface of the skull. The virus expression vector AAV-hSyn-GCaMPs (300 nl) was slowly injected (60 nl/min) unilaterally into the PBN area through a fine glass pipette (1-mm glass stock, tapering to a 10–20 micron tip). The pipette was kept in place for 10 min to allow the diffusion of the virus and was then slowly withdrawn. A microsyringe pump (Legato^®^ 130, KD Scientific, United States) was used for the injection. The coordinates of PBN were AP = -9.1 mm, ML = ± 1.8 mm, and DV = -7.3 mm, according to the rat brain atlas (Paxinos and Watson, 2007). After the injection, an optical fiber (200 μm O.D., 0.37 numerical aperture, Newton Inc., Hangzhou, China) was placed 100 μm above the injection site and fixed on the skull with a skull-penetrating screw and dental acrylic.

Fluorescence signals of GCaMPs were acquired by a multichannel fiber photometry system (Thinker Tech Nanjing Bioscience Inc, China) equipped with a 480-nm excitation LED (3W, CREE) and a dichroic mirror (DCC3420M; Thorlabs). An optical fiber (Newton Inc, China) integrated with an optical commutator (Doric Lenses) was used to guide the light between the fiber photometry system and the implanted optical fiber. The optical power at the tip of the fiber was set to 20–30 μW to minimize bleaching. The fluorescence signals of GCaMPs were filtered at 40 Hz and digitalized at 500 Hz and then recorded by the recording software (Thinker Tech Nanjing Bioscience Inc.).

For the propofol group, a catheter was first established on the caudal vein under isoflurane anesthesia. Recording started after an hour of washout of isoflurane. Following 100-s baseline recording, a single dosage of propofol (11 mg/kg) (Fu et al., 2017) was given through the caudal vein channel. For the isoflurane group, 1.4% isoflurane with oxygen at 1 L/min was applied. The moment of loss of righting reflex (LORR) and recovery of righting reflex (RORR) was marked and the recording was stopped 200 s after RORR. Five rats were assigned to each group and each anesthetic was tested three times with a 3-day rest period between experiments. All rats were scarified and subjected to immunofluorescence to verify viral expression and optical fiber implantation after the experiments were performed.

Fiber photometry data were exported to MATLAB 2016a (MathWorks, Cambridge, United Kingdom) for analysis. The change in the fluorescence (ΔF/F) was calculated by using (F–F_0_)/F_0_, where F is the test fluorescence signal, and F_0_ is the mean and standard deviation of the basal signal. The data were processed as previously described (Li et al., 2016; Zhou et al., 2017).

### Activation of PBN Neurons by DREADDs

Sixteen rats were assigned in each anesthetic group (propofol group and isoflurane group). A virus expression vector, either AAV-hSyn-hM3Dq-EGFP or AAV-hSyn-EGFP, was delivered bilaterally into the PBN area following the method described above (*n* = 8 for each group); clozapine N-oxide (CNO) (MedChemExpress) 1 mg/ml (dissolved in saline) at a dose of 5 mg/kg (Yu et al., 2015; Snow et al., 2017; Zhong et al., 2017) was injected intraperitoneally to activate the hM3Dq receptors 1 h before anesthetic application. Saline injections were used as controls. The behavioral tests and EEG recordings were conducted between 3 pm and 7 pm. To reduce the number of rats used in the experiment, each animal was tested multiple times with at least a 5-day washout between experiments. All efforts were made to minimize the number of rats used.

### Behavioral Testing

To quantify the induction and recovery time of propofol (Figure 3A), 50 min after CNO or saline injection, rats were acclimated in a Plexiglas chamber for 10 min, followed by continuous intravenous infusion of propofol (48 mg/kg/h) (Vanini et al., 2014; Fu et al., 2017). The induction time (also referred as LORR time) was determined by the interval between the beginning of the propofol infusion and the time when the rat lost its righting reflex for more than 30 s. For assessing the recovery time, propofol (48 mg/kg/h) was continued for a total of 40 min to assure an equilibrated concentration of propofol in the brain (Dutta et al., 1997). Rats were then placed in a supine position immediately after cessation of propofol. The recovery time (also referred as RORR time) was determined as the total duration between the stop of propofol infusion and the time when the rat resumed a prone position with four feet righting on the floor. The rats were maintained in the chamber during the induction, maintenance, and recovery periods.

For the isoflurane experiments, rats were placed in a sealed Plexiglas chamber filled with 1.4% isoflurane with oxygen at 1 L/min at 1 h after CNO or saline injection (Figure 3D). LORR time was determined by the interval between the onset of isoflurane treatment to the time when the rat demonstrated the loss of its righting reflex for more than 30 s. The anesthesia was maintained with 1.4% isoflurane for 30 min. Immediately after the cessation of isoflurane, the rats were gently taken out of the chamber and placed in a supine position in the room air to quantify the RORR time that was determined as the total duration between the cessation of isoflurane and the time when the rat resumed a prone position with its four feet righting on the floor.

### Implantation of EEG Electrodes

The surgery for electrode mounting was performed right after the injection of the virus expression vector AAV-hSyn-hM3Dq-EGFP (*n* = 12, 6 for each anesthetic group). Three screw electrodes for EEG recording were implanted into the skull as previously described (Kortelainen et al., 2012). Briefly, two recording electrodes were implanted 1.5 mm anterior to the bregma symmetrically over the left and right frontal lobes, 3 mm from each other, and a reference electrode was placed sagittally in the midline 1.5 mm posterior to the bregma. Dental acrylic and two skull screws were used to fix the EEG electrodes.

### EEG Recording and Analyses

After 1 h of CNO or saline injection, rats were anesthetized and connected with EEG cables. For the propofol test, a loading dose of 11 mg/kg propofol was injected intravenously for rapid induction of anesthesia, followed by continuous intravenous infusion of propofol (48 mg/kg/h) to maintain the anesthesia for 30 min (Figure 4A). For the isoflurane test, 1.4% isoflurane with oxygen at 1 L/min was administrated to induce LORR and then continued for 30 min (Figure 5A). EEG signals were recorded from the beginning of administration of anesthesia to emergence from general anesthesia.

EEG signals were amplified by a model-3000 amplifier (A-M Systems, United States) and collected by a CED Power1401-3 device (Cambridge Electronic Design, Cambridge, United Kingdom). The signals were filtered between 0.1 and 300 Hz. Data were digitized and recorded using the Spike2 software package (Cambridge Electronic Design, Cambridge, United Kingdom).

Spectrograms were constructed using multitaper methods implemented by the Chronux toolbox in MATLAB 2016a (MathWorks, Cambridge, United Kingdom) (Babadi and Brown, 2014). Each spectral density had a frequency range of 0 to 60 Hz, a window size of 5 s, and a band width of 2 Hz, and was computed using 9 tapers. Power spectrum analysis was conducted on the data from the period of anesthesia (5 min before cessation of anesthetic) and the recovery period (5 min after cessation of anesthetic). Delta, theta, alpha, beta, gamma, and total spectral powers were calculated using the frequency bands 1–4, 5–8, 9–12, 13–25, 26–60, and 1–60 Hz, respectively. Relative powers were calculated by dividing the averaged signal power across the frequency range of each band by the total power from 1 to 60 Hz.

### Perfusion and Immunofluorescence

After the completion of behavioral and EEG testing, rats with hM3Dq receptor (*n* = 6) were administered an injection of CNO (1 mg/ml, 5 mg/kg, i.p.) or saline (0.9%) (5 mg/ml, i.p.); then the rats were kept in their home cage for 2 h before perfusion. There was no other operation for rats during this time and all experiments were conducted in the light phase.

Rats were deeply anesthetized with 7% chloral hydrate for the perfusion of 0.01 M PBS followed by 4% PFA. The brains were harvested, post-fixed overnight at 4°C, and cryoprotected in 30% sucrose in PBS at 4°C until they sank. The brains were coronally sectioned into 30-μm slices on a cryostat (Leica CM1950).

For c-Fos staining, the brains sections were first incubated in a blocking solution (PBS containing 2.5% normal goat serum, 1.5% bovine serum albumin and 0.1% Triton^TM^ X-100) for 2 h at room temperature, incubated with the primary antibody (rabbit anti-cFos, 1:1000, Synaptic Systems) in a blocking solution overnight at 4°C, followed by a 3 min × 10 min wash with PBST (PBS with 0.1% Triton X-100, vol/vol). Sections were then incubated with the secondary antibody (goat anti-rabbit conjugated to Alexa 594, 1:1000 dilution, Invitrogen) at room temperature for 2 h. After another 3 min × 10 min wash with PBST, the sections were mounted on glass slides and cover-slipped with a mounting media (Gold antifade reagent with DAPI, Life Technologies, United States). Images of c-Fos immunostaining were captured on an Olympus BX63 virtual microscopy system.

The c-Fos-positive cells were counted on alternate sections on both sides of the brain in the PBN nucleus (approximately from bregma -8.88 to -9.48 mm), prefrontal cortex (PFC) (approximately from bregma 0.36 to -0.24 mm), BF (approximately from bregma 0.00 to -0.96 mm), lateral hypothalamus (LH) (approximately from bregma -2.40 to -3.48 mm), thalamus (TH) (approximately from bregma -2.16 to -3.12 mm), and supramammillary nucleus (SUM) (approximately from bregma -4.36 to -4.68 mm) (*n* = 6, 2–4 sections per rat).

### Statistical Analysis

The data are presented as mean ± SEM. Statistical analysis was performed and graph outputs were obtained with the GraphPad Prism software package, version 6.0 (GraphPad Software Inc., San Diego, CA, United States). Differences in calcium signals between the pre- and post-events were analyzed using paired Student’s *t*-tests. Unpaired Student’s *t*-tests were applied in the analysis of c-Fos expression between groups. Behavior results (LORR and RORR time) and EEG changes in the power spectrum were analyzed by two-way ANOVA followed by a Bonferroni *post hoc* test where appropriate. In all cases, a *p* < 0.05 was considered significant.

## Results

### PBN Neurons Are Activated During Emergence of General Anesthesia

**FIGURE 1:**
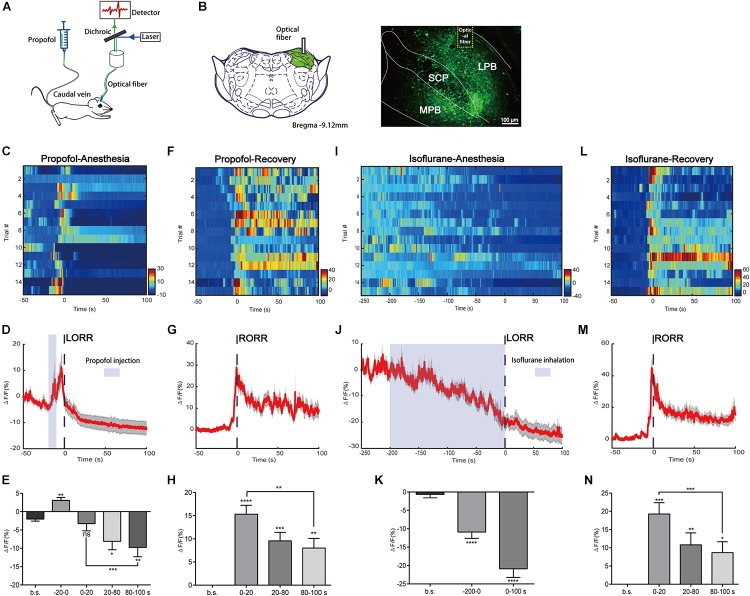
Neural dynamics of parabrachial nucleus (PBN) in response to propofol and isoflurane. **(A)** Scheme of fiber photometry setup. **(B)** Expression of GCaMPs in PBN neurons. Dashed yellow lines indicate the position of optical inserts. (Scale bars: 100 μm.) **(C–E)** Ca^2+^ signals associated with propofol-induced loss of righting reflex (LORR). Heatmap illustration of Ca^2+^ signals aligned to the initiation of LORR trails. Each row plots one trail and a total of 15 trials are illustrated. Color scale at the right indicates ΔF/F **(C)**. Peri-event plot of the average Ca^2+^ transients. Thick lines indicate the mean and shaded areas indicate SEM **(D)**. Statistical chart of changes in Ca^2+^ signals in propofol-induced unconsciousness **(E)**. ΔF/F represents change in GCaMPs fluorescence from the mean level before the propofol is given. b.s. represents baseline, which is the averaged ΔF/F between *t* = –50 and –20 s. (–20 to 0 s) represents the averaged ΔF/F from propofol injection to LORR. 0 represents the moment of LORR. (0–20 s), (20–80 s), and (80–100 s) represent the averaged ΔF/F in anesthesia period between *t* = (0–20 s), *t* = (20–80 s), and *t* = (80–100 s), respectively. **(F–H)** Ca^2+^ signals associated with emergence from propofol anesthesia. 0 represents the moment of RORR. b.s. represents baseline, which is the averaged ΔF/F between *t* = –50 and 0 s. The PBN neurons were activated during emergence from propofol anesthesia. Ca^2+^ signals associated with LORR **(I–K)** and emergence **(L–N)** from isoflurane anesthesia (*n* = 5). Same conventions as in the propofol group. PBN neurons were inhibited in the process of isoflurane anesthesia and activated in the emergence period. All data are shown as mean ± SEM. The *p*-values, compared with baselines or (0–20 s), are calculated by using paired *t*-test. ^∗^*p* ≤ 0.05; ^∗∗^*p* ≤ 0.01; ^∗∗∗^*p* ≤ 0.001; ^∗∗∗∗^*p* ≤ 0.0001; ns, not significant. LPB, lateral parabrachial nucleus; SCP, superior cerebellar peduncle; MPB, medial parabrachial nucleus.

To record the neuronal activity of the PBN under general anesthesia, we expressed GCaMPs into the PBN via the AAV-hSyn-GCaMPs virus vector. GCaMPs is a calcium indicator widely used to image neuronal activity. The *in vivo* Ca^2+^ signals indicated by the fluorescence of GCaMPs were recorded by calcium fiber photometry system under anesthesia (Figure 1A). The GCaMPs expression in the PBN was shown in Figure 1B.

In the group of propofol induced LORR, we analyzed Ca^2+^ signals in five time sections: baseline (wake: -50 to -20 s), the moment from propofol delivery to loss of reaction (-20 to 0 s), early period of anesthesia (0–20 s), and anesthesia period 1 (20–80 s) and 2 (80–100 s) (Figures 1C–E). It takes approximately 20 s for propofol injection to induce LORR; therefore, we set 20 s before LORR (-20–0 s) as the period from the moment propofol was given to loss of reaction. The Ca^2+^ signal acutely increased after propofol injection (*p* = 0.0016, paired *t*-test) that may be due to the acute pain sensation caused by propofol injection (Yuan et al., 2016). A significant decrease of the Ca^2+^ signal was noted in anesthesia period 1 and 2; however, no significant decrease of Ca^2+^ signal was observed during the early period of anesthesia (0–20 s) (*p* = 0.5705, paired *t*-test), suggesting that the PBN neurons are suppressed in the anesthesia phase but may not be an initial decisive factor for propofol-induced unconsciousness. In the emergence period, we observed a robust increase in PBN calcium signals related to the moment of RORR (Figures 1F, G). Four time sections were analyzed, including baseline (-50–0 s), early emergence period (0–20 s), and emergence period 1 (20–80 s) and 2 (80–100 s). Ca^2+^ signals in all emergence periods showed a significant increase as compared to baseline, especially in the early emergence period (*p* < 0.0001, paired *t*-test, Figure 1H). After the early emergence period, a decrease was seen following emergence time (0–20 s: 15.31 ± 1.935% vs. 80–100 s: 8.01 ± 2.081%; *p* = 0.0064, paired *t*-test). These findings indicate that PBN neurons were robustly activated during emergence from propofol anesthesia.

We also analyzed the changes in Ca^2+^ signals in isoflurane anesthesia (Figures 1I–N). It takes approximately 200 s for isoflurane to induce LORR; therefore, we set 200 s before LORR (-200–0 s) as the induction period. The Ca^2+^ signal gradually declined during isoflurane use, indicating that the neural activity of PBN neurons were inhibited by isoflurane. Similar to the propofol test, PBN neurons showed a significant increase in Ca^2+^ signals in response to emergence from isoflurane anesthesia (0–20 s, Δ*F*/*F*_0_ = 19.26 ± 3.1%, mean ± SEM; Figure 1N), while after the recovery of righting reflex, there was a decrease in Ca^2+^ signals (0–20 s vs. 80–100 s, *p* = 0.0002, paired *t*-test).

Taken together, these results show that PBN neurons are suppressed during anesthesia and are responsible for emergence from both propofol and isoflurane anesthesia, suggesting that PBN neurons may play a role in the recovery period.

### PBN Activation Accelerates Emergence From General Anesthesia

**FIGURE 2:**
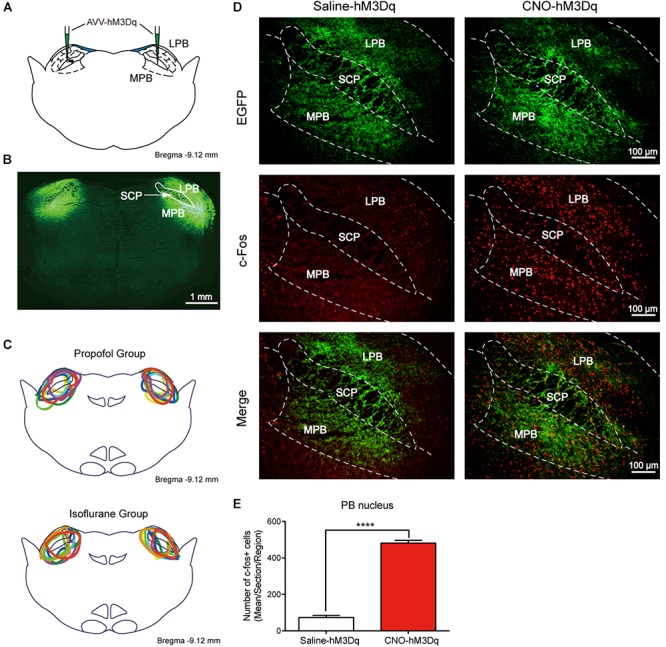
Expression of the hM3Dq receptors in PBN neurons and activation of neurons with CNO. **(A)** Coronal section showing the injection sites of AAV-hSyn-hM3Dq-EGFP or AAV-hSyn-EGFP in the PBN. **(B)** Outline of hM3Dq-EGFP in PBN nucleus. **(C)** Expressions of hM3Dq receptors in PBN from each case are outlined in the propofol group (*n* = 8) and the isoflurane group (*n* = 8). **(D)** c-Fos expression in PBN neurons after hM3Dq rats received CNO or saline i.p. injection. Three rats for each group (2–4 sections per rat). Bar = 100 μm. **(E)** Mean number of c-Fos positive cells in PBN nucleus. Data are described as mean ± SEM. c-Fos expression in PBN neurons with CNO pretreatment was significantly higher than in the saline pretreatment group (*p* < 0.0001, unpaired *t*-test). LPB, lateral parabrachial nucleus; SCP, superior cerebellar peduncle; MPB, medial parabrachial nucleus.

The higher level of activity in PBN neurons during the recovery phase suggests that the PBN might be important for recovery from anesthesia. To test this, we employed the DREADDs system to activate PBN neurons. In this system, hM3Dq fused with enhanced green fluorescent protein (EGFP) under human synapsin1 was introduced into PBN neurons via a virus expression vector (AAV-hSyn-hM3Dq-EGFP, titer >10^12^ genomic copies/mL, BrainVTA) (Figures 2A,B). The hM3Dq is an engineered muscarinic receptor that specifically responds to CNO to depolarize neurons (Armbruster et al., 2007). The extent of hM3Dq-EGFP expression in the PBN from each case is outlined in the propofol group (*n* = 8) and the isoflurane group (*n* = 8) (Figure 2C). C-fos, a neural activity marker (Carter et al., 2017), was applied to verify that the PBN neurons were activated by CNO. As shown in Figures 2D,E, CNO injection dramatically increased c-Fos expression in hM3Dq-expressing PBN neurons (*p* < 0.0001, unpaired *t*-test).

As CNO was reported to convert to clozapine *in vivo*, and may result in a false-positive outcome (Gomez et al., 2017), we set AAV-hSyn-EGFP-infected rats treated by either saline or CNO as additional control groups. No significant difference was found between saline- and CNO-treated control groups in both propofol and isoflurane tests, indicating that CNO does not activate PBN without the expression of hM3Dq receptor in our experiment settings.

**FIGURE 3:**
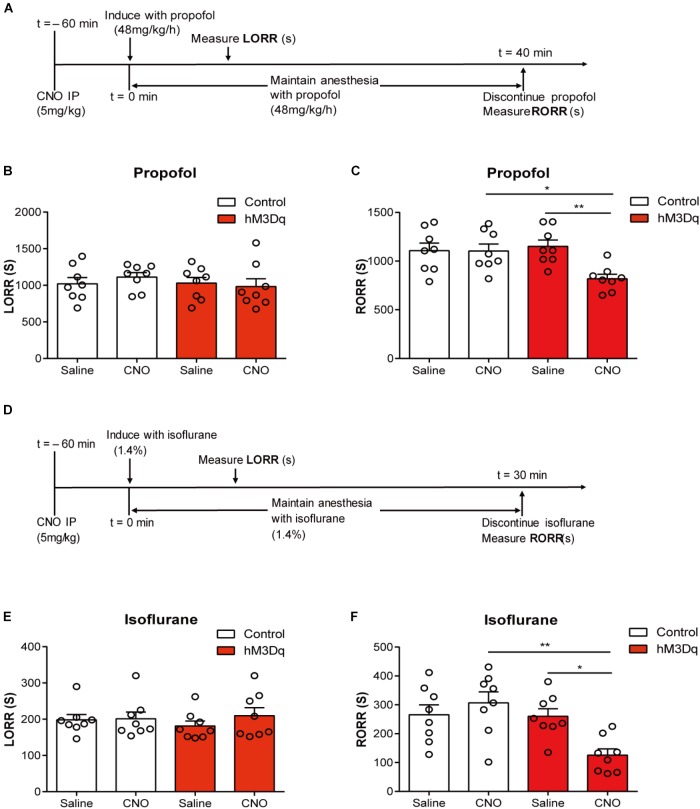
PBN activation accelerates emergence from general anesthesia. **(A)** Timeline for quantifying changes in induction and recovery time with propofol. **(B)** Results of propofol inducing LORR time in the control group (AAV-hSyn-EGFP) and the hM3Dq group (AAV-hSyn-hM3Dq-EGFP), both of which received saline or CNO injection respectively. The hM3Dq animals injected with CNO do not show a significant difference (*p* = 0.4214, *F*_1,28_ = 0.6659, two-way ANOVA) in induction time relative to each of the other three groups. **(C)** The hM3Dq animals injected with CNO show a significant decrease in recovery time relative to hM3Dq-saline rats (*p* = 0.0066) and control-CNO rats (*p* = 0.0103) in propofol anesthesia (Bonferroni’s *post hoc* test after two-way ANOVA). **(D)** Timeline for quantifying changes in induction and recovery time with isoflurane. **(E)** The hM3Dq animals injected with CNO do not show a significant difference (*p* = 0.1844, *F*_1,28_ = 1.852, two-way ANOVA) in induction time of isoflurane anesthesia. **(F)** The hM3Dq rats injected with CNO show a significant decrease in recovery time relative to hM3Dq-saline rats (*p* = 0.0263) and control-CNO rats (*p* = 0.0016) in isoflurane anesthesia (Bonferroni’s *post hoc* test after one-way ANOVA). All summary graphs show mean ± SEM; *n* = 8 for each group. ^∗^*p* < 0.05 and ^∗∗^*p* < 0.01.

Activating the PBN did not alter the induction time in both propofol (*F*_1,28_ = 0.4214; *p* = 0.6659; two-way ANOVA; *n* = 8; Figure 3B) and isoflurane tests (*F*_1,28_ = 1.8520; *p* = 0.1844; two-way ANOVA; *n* = 8; Figure 3E). However, the activation of PBN accelerates the recovery from propofol anesthesia; a significant difference of recovery time was found between the hM3Dq-CNO group and the hM3Dq-saline group (818.6 ± 45.51 s vs. 1151 ± 62.21 s, *p* = 0.0066), as well as between the hM3Dq-CNO group and the control-CNO group (818.6 ± 45.51 s vs. 1104 ± 71.41 s, *p* = 0.0103) (Figure 3C), indicating that the PBN neural activation leads to a faster emergence from propofol anesthesia. Similarly, the activation of PBN also shortened the recovery time of isoflurane anesthesia; the hM3Dq rats injected with CNO (125.5 ± 21.85 s) show a significant decrease in recovery time relative to the hM3Dq-saline-treated rats (260.4 ± 26.28 s, *p* = 0.0263) and the control-CNO rats (306.8 ± 37.96 s, *p* = 0.0016) (Figure 3F). These findings demonstrate that the activation of PBN promotes the behavioral arousal from both propofol and isoflurane anesthesia.

### PBN Activation Drives Cortical Arousal From General Anesthesia

**FIGURE 4:**
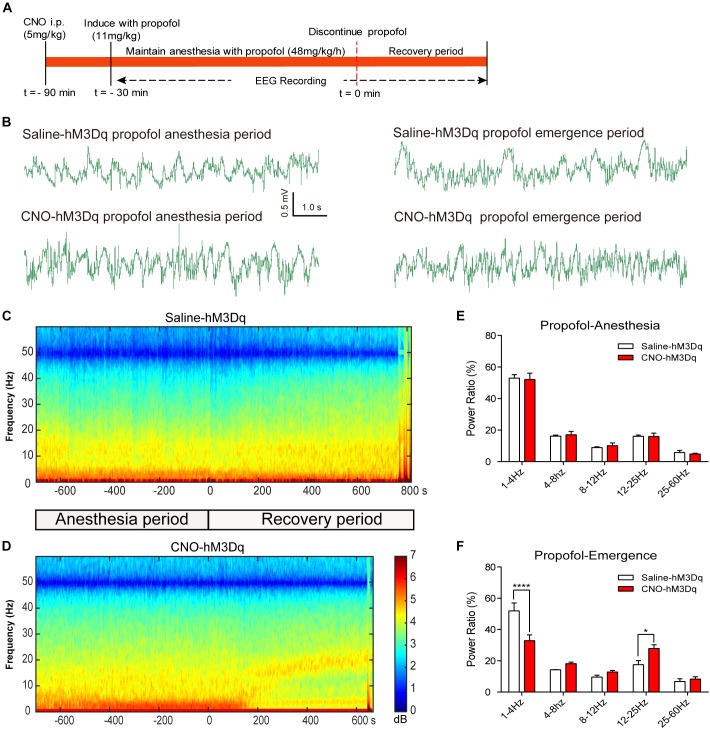
EEG changes under PBN activation in propofol anesthesia. **(A)** Timeline for EEG recording in propofol anesthesia. **(B)** Representative EEG traces during anesthesia and emergence period in hM3Dq rats pretreated with CNO or saline. **(C,D)** Spectrograms of EEG power during propofol anesthesia and emergence period after delivery of saline **(C)** or CNO **(D)** i.p. 0 on the time axis indicates the cession of propofol infusion. Warm colors (e.g., red) represent higher power at a given frequency, whereas cool colors (e.g., blue) represent lower power. **(E)** In the propofol anesthesia period, EEG traces 5 min before cessation of anesthetic were compared. No significant difference was found between CNO and saline use in any of the frequency bands quantified (*F*_4,50_ = 0.1241, *p* = 0.9731, two-way ANOVA). **(F)** In the recovery period, power spectrum analysis was conducted on data from propofol infusion stop to 5 min after that. CNO i.p. injection in hM3Dq rats shows a significant difference compared to the hM3Dq-saline group, displaying a significant decrease in δ power (1–4 Hz) and an increase in β power (12–25 Hz). All summary graphs show mean ± SEM (*n* = 6); ^∗^*p* < 0.05 and ^∗∗∗∗^*p* < 0.0001, Bonferroni’s *post hoc* test after two-way ANOVA.

**FIGURE 5:**
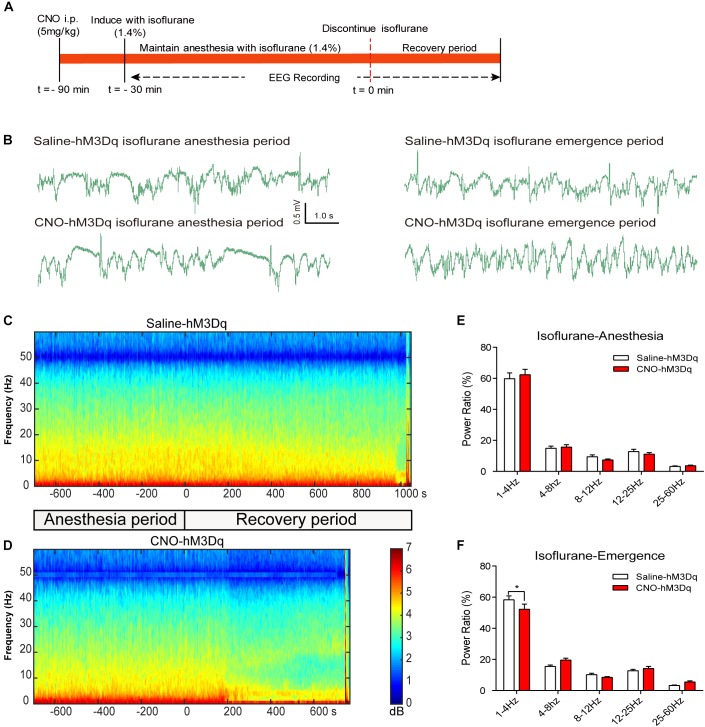
EEG changes under PBN activation in isoflurane anesthesia. **(A)** Timeline for EEG recording in isoflurane anesthesia. **(B)** Representative EEG traces during anesthesia and emergence period in hM3Dq rats pretreated with CNO or saline. **(C,D)** Spectrograms of EEG power during isoflurane anesthesia and emergence period after delivery of saline **(C)** or CNO **(D)** i.p. 0 on the time axis indicates the cession of isoflurane. **(E)** In the isoflurane anesthesia period, EEG traces 5 min before cessation of anesthetic were compared. No significant difference was found between CNO and saline use in any of the frequency bands quantified (*F*_4,50_ = 0.4886, *p* = 0.7441, two-way ANOVA). **(F)** In the recovery period, power spectrum analysis was conducted on data from isoflurane inhalation stop to 5 min after that. CNO i.p. injection in hM3Dq rats, compared to the hM3Dq-saline group, shows a significant decrease in δ power (1–4 Hz). All summary graphs show mean ± SEM (*n* = 6); ^∗^*p* < 0.05; Bonferroni’s *post hoc* test after two-way ANOVA.

To further evaluate how the activation of PBN neurons could affect arousal, we performed EEG recording over the corresponding cortical area in arousal. Figure 4 shows no difference of EEG activity between the CNO-hM3Dq and saline-hM3Dq groups during the propofol anesthesia period (*F*_4,50_ = 0.1241, *p* = 0.9731, *n* = 6, two-way ANOVA). However, after propofol infusion stopped, a quick transition from an anesthesia-EEG state to a wake-EEG state was observed in the PBN-activated rat group (Figures 4C,D). Spectral analysis of EEG data at 5 min after propofol stop shows a significant interaction between CNO and EEG spectral frequency (*F*_4,50_ = 10.38; *p* < 0.0001, *n* = 6, two-way ANOVA). Specifically, the activation of PBN neurons by CNO leads to a significant decrease in δ band (1–4 Hz) power and a significant increase in β band (12–25 Hz) power, while 𝜃 (4–8Hz), α (12–25Hz), and γ (25–60Hz) band power remain unchanged (Figure 4F), indicating a wake transition as described before (Purdon et al., 2015). Figure 5 shows the EEG result of the isoflurane test. Similar to the propofol test, the activation of PBN had no effect on the EEG spectral power ratio during the anesthesia period (*F*_4,50_ = 0.4886, *P* = 0.7441, *n* = 6, two-way ANOVA; Figure 5E). However, during the emergence period, a significant change was also observed in the PBN-activated rat group (Figures 5C,D). Spectral analyses show that the activation of PBN decreases δ band power (*p* = 0.0401, *n* = 6, Bonferroni’s *post hoc* test) (Figure 5F), consistent with a previous report that electrical stimulation of PBN nucleus led to a significant decrease in δ power and an increase in 𝜃 power in isoflurane anesthesia (Muindi et al., 2016). Thus, the EEG data confirm that the activation of PBN drives cortical arousal after general anesthesia with both propofol and isoflurane.

### PBN Activation Increases c-Fos Expression in Arousal-Related Area

**FIGURE 6:**
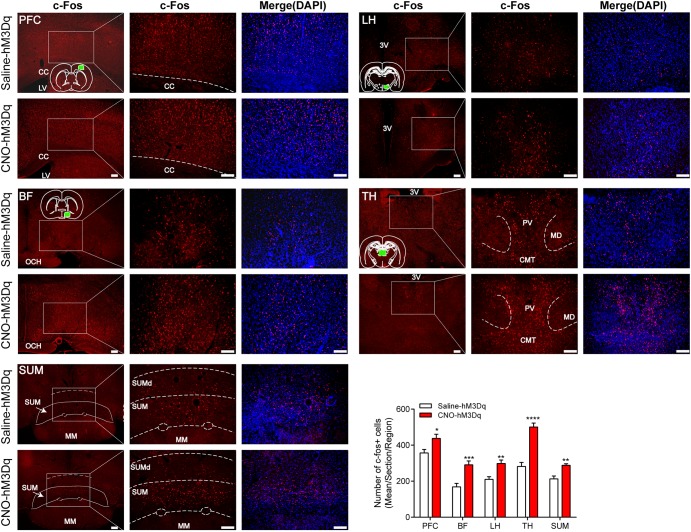
c-Fos expression in prefrontal cortex (PFC), basal forebrain (BF), lateral hypothalamus (LH), thalamus (TH), and supramammillary nucleus (SUM) 2 h after hM3Dq rats received CNO or saline. Green box in the standard rat brain map indicates the part where we counted. The PBN activation group shows an increase in c-Fos expression compared to the PBN non-activation group in these five areas. Data are expressed as mean ± SEM. Unpaired Student’s *t*-tests are used for comparisons. ^∗^*p* < 0.05, ^∗∗^*p* < 0.01, ^∗∗∗^*p* < 0.001 and ^∗∗∗∗^*p* < 0.0001. Bar = 100 μm. LV, lateral ventricles. 3V, the third ventricle. OCH, optic chiasm. PV, paraventricular thalamic nucleus; MD, mediodorsal thalamic nucleus; CMT, central medial thalamic nucleus; SUM, supramammillary nucleus; SUMd, supramammillary decussation; MM, mamillary nucleus.

To test downstream area activated after the PBN neural activation, we examined c-Fos expression in areas projected by PBN neurons. Figure 6 shows that the activation of PBN elicits a significant increase in c-Fos expression in the PFC (*p* = 0.0146), BF (*p* = 0.0004), LH (*p* = 0.0015), TH (*p* < 0.0001), and SUM (*p* = 0.0066), suggesting that the activation of PBN might act as a starting point to initiate a neural network in modulating the arousal from anesthesia.

## Discussion

Our study reveals a significant increase in the activity of PBN neurons during emergence from propofol and isoflurane anesthesia. The neuronal activation of PBN had no impact on the induction but accelerated the emergence and the cortical arousal from general anesthesia. The activation of arousal areas by the activation of PBN suggests that it may trigger an arousal network to promote wakefulness.

Calcium fiber photometry is one of the most sensitive and easiest ways to record neuronal activities of deep brain structures in living animals (Guo et al., 2015). It has been successfully applied to record event-related neuronal activities such as reward-related serotonin neurons in the dorsal raphe nucleus (Li et al., 2016) and cold responsive neurons in the dorsomedial hypothalamus (Zhao et al., 2017). The quick transition of anesthesia stages prompted us to take advantage of this sensitive system to identify the neuronal activity in the PBN. Using a calcium fiber photometry system, we recorded the activity changes of PBN neurons during the induction and emergence of anesthesia. Interestingly, the propofol injection through the caudal vein immediately elicited an abrupt increase, other than decrease in Ca^2+^ signaling in PBN neurons, which was possibly caused by the injection pain commonly seen in propofol application (Yuan et al., 2016), as PBN neurons are crucial for itch and pain signaling (Missig et al., 2014; Mu et al., 2017). Moreover, there was no increase in isoflurane induction. Compared with the results from the awake stage, no decline of Ca^2+^ signaling was detected in the first 20 s of the anesthesia period. These results suggest that propofol-induced LORR may not require a complete inhibition of PBN neurons. In the recovery period, from either propofol and isoflurane anesthesia, a distinct RORR responsive Ca^2+^ signaling peak was seen in PBN, indicating that PBN was highly activated at the emergence moment. Our results are consistent with a previous study that reported an increase in c-Fos expression in the PBN during emergence from isoflurane-induced anesthesia (Muindi et al., 2016).

The DREADDs strategy has been successfully applied in the activation of PBN neurons in sleep research (Qiu et al., 2016). With this strategy, we explored the function of PBN in general anesthesia. Contrary to our expectation, the activation of PBN neurons did not prolong the induction time in either propofol or isoflurane anesthesia, consistent with the EEG result that showed no difference of cortical activity between PBN-activated and non-activated rats during anesthesia. These results suggest that the inhibition of PBN neurons is not critical for anesthetics to induce the LORR. During the recovery period, the activation of PBN led to arousal from both propofol and isoflurane anesthesia, in accordance with the Ca^2+^ signaling and EEG changes. Consistent with this result, electrical stimulation of the PBN effectively induces the reanimation from isoflurane-induced anesthesia (Muindi et al., 2016). The EEG changes during emergence from isoflurane-induced anesthesia were less dramatic than from propofol-induced anesthesia. A possible reason is that the rats were kept in a sealed chamber for isoflurane administration. Even after the termination of the isoflurane flow, residual isoflurane in the chambers may still have been acting on the rats.

PBN is crucial for promoting arousal during the sleep–wake cycle (Qiu et al., 2016; Kaur et al., 2017). Our results supported the hypothesis that PBN also has an arousal-promoting role in general anesthesia. Moreover, by c-Fos immunostaining, we found a potential role for the PBN to initiate further activation of a wake-related network in the PFC, BF, LH, TH, and SUM. These results support the hypothesis that ascending projections from the PBN target the BF (Hu et al., 2016) and the LH (Niu et al., 2010) to maintain cortical arousal and behavioral wakefulness. The connection between these structures may contribute to wakefulness promotion of PBN, as both the BF and the LH contain wakefulness-promoting cell groups (Anaclet et al., 2015; Venner et al., 2016) and have proven to have key roles in the recovery from general anesthesia (Leung et al., 2014; Zhang et al., 2016). Moreover, SUM that has been reported as a key node of the arousal system (Pedersen et al., 2017) is also activated following the activation of PBN by DREADDs and may also act on wakefulness promotion of PBN. Interestingly, we also found that activation of PBN by DREADDs elicited a significant increase of c-Fos expression in the midline TH, including the paraventricular and central medial thalamic nucleus (Figure 6), which have been reported to play a role in modulating arousal (Colavito et al., 2015; Fu et al., 2017). Yet, the activation of PBN neurons by DREADDs has been functionally proved to drive cortical arousal independent of the TH (Qiu et al., 2016), and the activation of glutamatergic thalamocortical neurons by DREADDs produced robust thalamic c-Fos activation in the intralaminar and midline nuclei, but did not drive wakefulness (Anaclet et al., 2015). Therefore, it appears that these thalamic regions might be not functionally important in the wakefulness promotion of PBN. However, these results indicate that the activation of PBN could further activate some downstream areas, but a further functional study is required to resolve whether these c-Fos changes are important for PBN activity-induced arousal.

In addition to its role in modulation of the arousal system, the PBN area also functions in the transmission of sensations, particularly pain (Roeder et al., 2016) and itching (Mu et al., 2017). As seen in the data obtained from calcium fiber photometry, propofol injection led to an abrupt increase in the Ca^2+^ signal, indicating that a pain-like stimulus could lead to the activation of PBN neurons. This may explain why during the recovery period from anesthesia, a stimulus like pain could decrease the time to emergence. This may be similar to our observations during clinical work, where we rouse patients in the post-operative period by calling out their names or lightly tapping them on their shoulders, to try to wake them up more quickly and potentially reduce complications after the surgery has been completed.

There has been a growing interest in the role of ascending arousal pathways in modulating general anesthesia. Optogenetically activating dopamine neurons in the ventral tegmentum has been shown to induce behavioral and cortical arousal as well as the RORR from general anesthesia (Taylor et al., 2016). Using the DREADDs method, a previous study found that activation of the noradrenergic system from the locus coeruleus strongly prolonged induction time and accelerated emergence from isoflurane anesthesia (Vazey and Aston-Jones, 2014). Together, the evidence from our study and prior work by others suggests that the ascending arousal pathways play a role in modulating general anesthesia. Pharmacologically manipulating these arousal systems may provide a new approach to accelerating recovery from general anesthesia.

The present work adds to the growing body of evidence that emergence from surgical anesthesia is not a simple process. It represents a reversal of induction, and is in agreement with the study of Kelz *et al* suggesting that the neural substrates involved in induction and recovery from anesthesia are different. Our previous study reported that microinjection of NE into the central medial thalamic nucleus accelerated emergence from propofol anesthesia but had no impact on the induction or the sensitivity to propofol anesthesia (Fu et al., 2017). Lesions of the ventral tegmental area did little to impact the induction time or ED50 of propofol but significantly prolonged the emergence time from anesthesia (Zhou et al., 2015). Similar effects were noted after isoflurane anesthesia in subjects with orexin-saporin lesions of the tuberomammillary nucleus (Luo and Leung, 2011). Consequently, induction and emergence from general anesthesia may not employ the same mechanisms to control the anesthesia-arousal cycle. However, it is still need to determine whether manipulation of specific neurons in the PBN could affect both induction time and recovery time.

The present study has some limitations. First, changes in respiration (Zuperku et al., 2017), cardiac activity (Davern, 2014), and thermosensation (Yahiro et al., 2017) that accompany induction and emergence from general anesthesia may also contribute to the results of calcium fiber photometry. Second, although we found that the activation of PBN increases c-Fos expression in the PFC, BF, LH, TH, and SUM, a further functional confirmation is needed. Finally, the PBN area contains a number of neuron populations. Non-specifically activating of PBN neurons does not address the function of specific neuron populations and presents the combined effects of all the stimulated neurons in the PBN nucleus. Selective techniques for further analysis on the function of specific neuron populations are needed.

In summary, these findings support the hypothesis that PBN neurons play a role in modulating emergence from general anesthesia.

## Author Contributions

WY and TY contributed to the conception and design of the study. YJ was responsible for the evolution of overarching research goals and aims. SY and SC performed the behavioral tests and c-Fos staining. YZ provided methodological support and revised analyzed data. TL was responsible for EEG, calcium fiber photometry, and analysis and drafting the manuscript.

## Conflict of Interest Statement

The authors declare that the research was conducted in the absence of any commercial or financial relationships that could be construed as a potential conflict of interest.
